# Nurses’ experiences of encountering patients with mental illness in prehospital emergency care – a qualitative interview study

**DOI:** 10.1186/s12912-022-00868-4

**Published:** 2022-04-18

**Authors:** Zetterberg Johanna, Visti Elin, Holmberg Mats, Andersson Henrik, Aléx Jonas

**Affiliations:** 1 Department of Ambulance Service, Region Sörmland, Eskilstuna, Sweden; 2 Faculty of Health and Life Sciences, Linnaeus University, Växjö, Sweden; 3 Centre of Interprofessional Collaboration within Emergency care (CICE), Linnaeus University, Växjö, Sweden; 4 Centre for Clinical Research Sörmland, Uppsala University, Eskilstuna, Sweden; 5 School of Health, Care and Social Welfare, Mälardalen University, Eskilstuna, Sweden; 6 Faculty of Caring Science, Work Life and Social Welfare, University of Borås, Borås, Sweden; 7 PreHospen – Centre for Prehospital Research, University of Bora °s, Borås, Sweden; 8 Department of Nursing, Umeå University, 901 87 Umeå, Sweden

## Abstract

**Background:**

Nurses working in prehospital emergency care (PEC) encounter patients with all types of health conditions. Increasingly, they are encountering patients suffering from mental illness and this trend reflects the worldwide increase in mental illness. There is very little current knowledge of encounters between nurses and patients with mental illness in ‘PEC’, especially from the nurses’ perspectives.

**Aim:**

The aim of the study is to investigate nurses’ experiences of encountering patients with mental illness in ‘PEC’.

**Methods:**

The participants were recruited in a region in southeast Sweden (that covers approximately 5600 km^2^ and has 300,000 inhabitants). In total, 17 nurses consented to participate. The participants were asked to narrate their individual experience of encountering patients with mental illness. The interviews were transcribed verbatim, then analysed with qualitative content analysis.

**Results:**

The result is presented in terms of three themes and eight sub-themes. The main themes are ‘Lacking trust in the patient and one’s own abilities’, ‘Being under internal and external influences’ and ‘Moving towards a genuine nurse-patient relationship’.

**Conclusion:**

The results show that nurses strive to lay the foundation for a trusting relationship. Simultaneously nurses encountering is characterized by a mistrust and it is influenced by pre-understanding and emotions when they take care for patients. The findings could be used to develop nurses’ readiness and capability to encounter patients with mental illness and to respond appropriately to the patients somatic and mental care needs.

## Background

Nurses working in prehospital emergency care ‘PEC’ encounter patients with all types of mental illness. In this article ‘Mental illness’ is used as an umbrella term covering severe disorders/diseases, common mental health problems, and mild symptoms of distress. Examples include eating disorders, schizophrenia, anxiety, bipolar disorder, substance use disorder, depression, self-harm, sleeping difficulties, and distress [1]. ‘PEC’ refers to the care and treatment provided by nurses in the emergency medical services (EMS). This care takes place outside formal health care institutions often in the patient’s home. ‘PEC’ is often provided by EMS ambulatory resources such as ambulances and single responder units. In this article nurses refer to register nurses with or without specialist education.

The nurses are expected to offer professional care and to assess each patient’s condition while showing respect for the patient’s self-determination and integrity [2]. Increasingly, they are encountering patients suffering from mental illness [3, 4]. This trend reflects the worldwide increase in mental illness [5]. In Sweden there has been a rise in mental illness in the population the last ten years [2].

Assignments involving patients with mental illness are common in ‘PEC’ [6], and nurses working in this service need enhanced educational support [7]. Earlier research has identified the complexity of assessing patients with mental illness in emergency care. It has also shown that nurses find such patients less interesting than patients with emergency somatic conditions [8]. Nurses were also found to need increased knowledge to be able to make the required assessments [9]. While cooperation with a psychiatric nurse has been found to be effective, nurses in ‘PEC’ also need to learn more about mental illness and the appropriate approach when assessing such patients [10].

In ‘PEC’, care and treatment should be person-centred [11]. A trusting nurse–patient encounter is also a core part of ‘PEC’ [12], and these encounters can be understood as a caring relationship. However, nurses have been found to dichotomize medical care and a caring relationship, with their primary focus being on medical care and treatment [13]. This attitude can affect their encounters with patients with mental illness who do not manifest explicit emergency somatic symptoms. Patients who seek care and treatment for mental illness do not always feel that they are treated with respect, and they feel stigmatized [14]. Another problem is that patients with mental illness feel that the nurse’s focus in the encounter is primarily on their somatic symptoms rather than on their mental health [15]. Hence, the patient may not request the care they need, and their condition may deteriorate, resulting in ‘PEC’ nurses later encountering patients with still more extensive care needs [16].

There is clearly a need for increased competence in assessing patients with mental illness in ‘PEC’ [7, 17]. Because a trusting nurse–patient encounter is important for a good care outcome, there is a need for additional knowledge and research in this area. There is currently little knowledge of encounters between nurses and patients with mental illness in ‘PEC’, especially from the nurses’ perspectives. Therefore, the aim of this study is to expand this base of knowledge from the nurses’ perspectives.

## Aim

The aim of the study is to investigate nurses’ experiences of encountering patients with mental illness in ‘PEC’.

## Methods

### Design

A qualitative inductive design was adopted for this study. Semi-structured interviews were used to collect data. To ensure the study’s rigor the COREQ-checklist [18] was used (see Appendix 1).

### Settings and participants

This study was conducted in southeast Sweden and covers approximately 5600 km^2^ and has 300,000 inhabitants. The EMS in the region has eight EMS stations, covering both rural and urban areas, and employs both nurses and emergency medical technicians. In 2019 the EMS had approximately 33,000 assignments.

A purposeful sampling was adopted [19]. Contact details for nurses who met the inclusion criteria i.e., nurses working in PEC, were provided by first-line managers to a research nurse in the project. Those nurses were emailed information about the study’s aim, procedures, and ethical aspects and were then asked by the research nurse whether they would consent to participate in the study. There were no exclusion criteria. In total, 17 nurses consented to participate. They included nurses of different ages, genders, years as a nurse, years in EMS and with or without specialist education (see Table 1). Three research nurses conducted individual interviews with the 17 nurses.

**Table 1:** Demographic and professional characteristics of the participating nurses

Number of participating nurses	17
*Women*	*9*
*Men*	*8*
**Age (years**)	
*30–39*	*8*
*40–49*	*8*
*50–59*	*1*
**Years as nurse**	
*Range*	*4–25*
*Median*	*10*
**Years in EMS**	
*Range*	*3–20*
*Median*	*7*
**Educational background**	
*Nurses without specialist education*	*8*
*Nurses with specialist education*	*9*
*One area (Ambulance or anesthetic care)*	*5*
*Two areas (Ambulance together with primary or anesthetic care)*	*2*
*Three areas (Ambulance, anesthetic and primary care)*	*1*

### Data collection

Data was collected through individual open-ended interviews conducted by three research nurses at the participants’ workplaces during spring and autumn in 2018. The interviews were based on a semi-structured interview guide designed to encourage the participating nurses to reflect on their experiences of encountering patients with mental illness. The interviews were introduced with an opening question: ‘Can you describe a situation involving a patient with mental illness, and how you handled the situation?’. Follow-up questions were posed where necessary to stimulate reflections on their experiences, including ‘What made this situation difficult?’ or ‘How did the conversation with the patient proceed?’ The interviewers’ experience from EMS may have affected their probing questions. The interview guide was pilot tested in the first interviews with no amendments deemed necessary. Each interview was digitally recorded and transcribed verbatim by a professional transcriber. The interviews encompassed a total of 446 min recorded data (range 15–39 min/interview, mean = 29 min). The transcribed data – as foundation for the following data analysis - filled 147 A4 pages with 1.5-line spacing.

### Data analysis

The data was subjected to a qualitative content analysis with an inductive descriptive approach, in accordance with Sandelowski [20]. This method was selected to highlight the ‘PEC’ nurses’ experiences and to put their stories into context [21]. The data analysis followed a triangulation process to ensure scientific rigor and trustworthiness of the study. Firstly, the transcribed interviews were read repeatedly (by the first and second author) as a whole text without listening to the recorded data and with notes being made in the margin to begin structuring the data. Subsequently, meaning units were selected that corresponded to the aim of the study. The meaning units were condensed and manually coded to describe the content with the aim of keeping the core content. Finally, the codes were grouped into larger sets to form themes and sub-themes. Generating themes and sub- themes was done in an ambition to identify patterns in the data and to increase the level of abstraction (see Table 2). To minimize the risk of misinterpretation based on the authors’ preunderstanding, the interviews were then read as a whole again. All authors had constant access to the transcribed material. The formulated sub-themes and themes were reviewed based on their message and meaning in workshops involving all the authors. In addition, in line with a triangulation process, the analysis was presented at a critical research seminar - with junior and senior researchers outside the research group - at the Centre of Interprofessional Collaboration within Emergency Care, Linnaeus University, before the final themes and sub- themes were agreed upon. The analysis continued throughout the whole writing process, moving back and forth between the transcribed data and the formulated themes.

**Table 2:** Data analysis, from meaning units to themes

Meaning unit	Condensed meaning unit	Code	Sub-themes	Themes
” … sometimes it can be easier talking to young people in these situations, not that they are necessarily better at listening, but it can be easier to say what you want to say and stuff”	Sometimes it can be easier to talk to young patients with mental illness than to older people. It is easier to get message across.	Easier to reach young patients with mental illness	Breaking down the patient’s barrier	Moving towards a genuine nurse-patient relationship
“on the kitchen work top lies a carving knife, first problem was like, wow, this is not the situation that we sort of could expect from the call”	On the kitchen work top there was a carving knife we didn’t expect from the call.	To be in a situation that doesn’t meet the expectations.	Being influenced by pre-understandings and emotions	Being under internal and external influences

Four of the authors had experience of ‘PEC’ and three had previous experience of nursing research. The whole research group were nurses with at least one year of additional specialist nursing education. The analysis was performed in Swedish and finally translated into English.

### Ethical considerations

Ethical considerations were considered throughout the process in line with the Declaration of Helsinki [22]. The project was approved in advance by the Regional Ethics Committee in Stockholm, Sweden (Reg. no 2018/005). All the participants gave their informed consent. They were informed that participation was voluntary and that they could withdraw at any time without stating a reason, and they were given the opportunity to ask questions.

## Results

The result is presented in terms of three themes and eight sub-themes (Table 3). The main themes are ‘Lacking trust in the patient and one’s own abilities’, ‘Being under internal and external influences’ and ‘Moving towards a genuine nurse-patient relationship’.

**Table 3:** Overview of sub-themes and themes

Sub-themes	Themes
Distrusting the patient	Lacking trust in the patient and one’s own abilities
Feeling inadequate	
Being influenced by pre-understandings and emotions	Being under internal and external influences
Being part of a multifaceted collaboration	
Establishing explicit boundaries and control	Moving towards a genuine nurse-patient relationship
Breaking down the patient’s barrier	
Being respectful and present	
Laying a foundation for a trustful relationship	

### Lacking trust in the patient and one’s own abilities

The participants described encounters with patients with mental illness as characterized by a lack of trust in both the patient and oneself. Patients with mental illness were experienced as unreliable, generating distrust among the nurses. However, this response was found to be complex and related to the nurses’ desire for genuine contact with the patients. Not being able to establish such contact led to a sense of inadequacy and hopelessness. Two sub-themes were identified, namely ‘Distrusting the patient’ and ‘Feeling inadequate’.

### Distrusting the patient

The participants experienced fear because the behaviour of patients with mental illness was unpredictable and situations could change quickly, becoming unsafe. Nurses therefore had to be on their guard, not fully trusting the patient. Being alone with the patient and not knowing whether the patient had insight into their mental illness created uncertainty. Attempts to establish contact with the patient could trigger a potentially dangerous situation by evoking threatening behaviour in the patient. So, the nurses experienced fear and insecurity.

He (the colleague) walks into the apartment and I wait outside in the stairwell cause I feel like I don ´t want to walk into this, but it is his … you got to take responsibility for yourself, and then all of a sudden he comes running out and after him runs a woman brandishing a pair of scissors … from a rather calm situation it suddenly becomes a critical situation and we are facing a death threat*.*

The information nurses received about a situation before arrival did not always correspond to what they encountered at the patient’s location. Therefore, they felt unprepared when, for example, a patient described as suffering from anxiety turned out to be threatening or aggressive. In such situations the patient’s behaviour did not correspond to the nurse’s expectations, and could suddenly change without warning. Nurses had to be prepared for unexpected patient reactions and had to adapt to changes under stressful conditions. The importance of early preparation for situations was underlined, as well as knowing what to expect and ways to work safely, such as good positioning in relation to the patient or calling in support from police officers to secure the situation. Other ways to create a sense of safety in unsecure patient encounters included being able to recognize the patient beforehand and working with a colleague with whom one was comfortable.

### Feeling inadequate

Nurses experienced feelings of inadequacy and hopelessness when, for example, they were unable to reach or protect the patient. This, in turn, caused them to doubt their professional competence and responsibility, leading to feelings of failure and guilt. Complex conflicts of interest were also described. For example, nurses felt intrusive when compelled to force a patient to receive care, but felt they had abandoned the patient if they did not do so. This was especially the case when there was confusion about whether the patient’s condition required psychiatric or somatic care.

Patients with no real physical damage fall between the cracks. There is nobody who will take responsibility for them. And you would think that, to me it doesn’t matter if it is a physical or mental harm, it hurts just as much. But just on this mentally, it, no, no*.*

Consequently, nurses found it difficult to reason clinically about the encounter, afraid that doing the wrong thing might worsen the patient’s mental illness. There were also fears of missing signs of severe somatic diseases or suicidal thoughts and behaviours.

### Being under internal and external influences

The participants’ responses showed that during encounters with patients with mental illness they were experiencing both internal and external influences. The nurses’ pre-understandings about the patient, their own emotions, and their collaboration with others had both negative and positive influences on the actual patient encounter. The theme was developed in terms of two sub-themes: ‘Being influenced by pre-understandings and emotions and ‘Being part of a multifaceted collaboration’.

### Being influenced by pre-understandings and emotions

Nurses’ preconceptions about specific patients based on previous encounters had an impact on the present encounter. Therefore, there was a risk of not taking the patient’s situation seriously, so contributing to their discomfort and adding to their suffering. In such encounters the patient’s mental illness was perceived as less important than time-critical somatic conditions. This attitude was expressed in comments like the following:

What can be difficult with this woman is, one, well, taking it seriously, but also that, shall I say, she, in some ways it almost feels as if ‘well come on now, do it properly’ so something comes out of it like*.*

The patients were occasionally perceived as uninterested in participating in their own care. This was frustrating, but was described as easier to handle if the patient had made a serious suicide attempt. Such situations required a direct nursing intervention to prevent severe outcomes, disregarding the patient’s own will. However, such encounters could be emotionally traumatic and draining.

When I got in the car, the thought hits me that the mum left her 1-year-old in the buggy outside, she said that she was going for a walk, leaves her 1-year-old in the buggy, goes upstairs and kills herself, and then I have got my 1-year-old daughter at home and there and then I decided that I will not be able to continue working this shift*.*

In such situations, nurses felt helpless – wanting to help the patient, but seemingly incapable of doing so. The nurses were emotionally affected by types of encounters that are difficult to get used to, including suicide attempts and self-harm. The emotional impact *of* such encounters could decrease their ability to finish a shift.

### Being part of a multifaceted collaboration

Cooperation was perceived as important in encounters with mentally ill patients, and police support was considered valuable. However, when cooperation failed, it was perceived as a result of being unprofessional and a failure. Sometimes personnel from the police or fire department could reach and calm a patient when the nurses could not. On the other hand, the presence of police or firefighters could become a distraction if the patient was perceived as losing their focus on the established nurse–patient encounter because several professionals were involved.

But maybe also that you have to call for the police, because things are different when they step into the room. It usually calms the situation or escalates it, but in this case, it would probably have calmed it down, it becomes reality somehow*.*

Functional collaboration in the ambulance team when encountering patients with mental illness is easier with a colleague who shares similar attitudes and values. If, however, a colleague expresses negative attitudes when reading the dispatch information before the patient encounter, these negative attitudes became an obstacle in the upcoming encounter. It becomes difficult to collaborate if the colleague has a different perception of the patient, or of whether the situation is to be regarded as threatening.

### Moving towards a genuine nurse–patient relationship

The participants stated that their goal during an initial encounter with a mentally ill patient was to lay the foundation for a trusting relationship. In order to accomplish this, they needed to break through the patient’s barriers, have explicit boundaries and control, as well as a respectful and present approach. This theme has four sub-themes: ‘Establishing explicit boundaries and control’, ‘Breaking down the patient’s barriers’, ‘Being respectful and present’ and ‘Laying a foundation for a trusting relationship’.

### Establishing explicit boundaries and control

The importance of establishing explicit boundaries related both to the nurse’s personal safety and the safety of the patient. Some encounters required a seemingly harsh approach, ranging from being very determined to using physical constraints. Nurses also described presenting alternative care options or demanding that the patient consider alternative responses as a way of establishing explicit boundaries and controlling the encounter. However, this required the nurses to be sensitive in assessing the alternatives in each unique patient encounter.

I will say: ‘You need help and if you come with me, we can go to the psychiatric clinic, where you will get help and then we‘ll go, you and me, and my colleague will drive and it will be all smooth, nice and calm, and we could sit and chat all the way and stuff and you can tell me. But otherwise, I would have to, yes well, then the police would have to come and get you, to help us get you to hospital and it will not be that good, I promise you.’

Encountering patients with mental illness required the nurses to have knowledge of how to proceed to provide the best possible care options for each patient’s unique situation. The nurses experienced their responsibility as having to take control of the situation so that things proceeded in a proper way. This required being like a ‘spider in a web’, with a general overview of the patient’s situation and different care alternatives.

### Breaking down the patient’s barriers

In encounters with patients suffering from severe mental illness such as suicidal behaviour or psychosis, the respondents often found the patient difficult to reach. This was described as the patient having a barrier. The nurses needed to break through these barriers to gain access to the patient. However, this was sometimes experienced as impossible as the barrier was too high. The nurses would then make fruitless attempts to calm and divert the patients by talking. However, this was experienced as difficult due the patient’s specific mental illness or their being unreceptive to communication.

Several times when I have been there, she has taken tablets … I try to talk with her, I mean when she has been awake, so I do try talking with her … or she is rather closed off, she wants to, is not so interested in talking. So, she is really difficult to reach, but I talk to her anyway*.*

Being able to encounter the patient with calmness and respect was described as essential. Doing so requires the ability to form some idea of what the patient is thinking. Active listening without interrupting was important. Children were described as being able to show trust and adapt to the nurses, but adults were described as demanding, requiring more thought and reflection in communication.

Nurses perceived patients with mental illness as being in crisis, and endeavoured to adapt to the patient’s situation by creating a calm encounter. Responsiveness and information-gathering are important aspects of such encounters. However, the presence of others (e.g., significant others, police officers or firefighters) could disrupt the encounter, and so the nurses sought to protect patients from such stressors. For example, they might ask people who might have a negative impact on both the patient’s mental illness and the establishment of a trusting caring relationship to leave the room.

### Being respectful and present

It is important that the nurse to be open, instructive, responsive and non-judgemental. This also involves showing respect for the patient’s surroundings, home and way of life. The involvement of relatives was described as a ‘bridge’ to reach the patient. Providing time for the encounter is fundamental, as well as daring to encounter the patient in the present.

A patient with mental health problems, it ´s having the patience that, that not just like if someone says ‘go to hell’, I have to understand and get that, that I definitely cannot leave this ill person behind but that is what, that is not what this person needs, this person needs help*.*

It takes patience to be able to understand the patient and not be provoked in the encounter. However, patience sometimes runs short, especially during a long shift with sleep deprivation. It was difficult encountering patients who did not understand their need for care due to a lack of insight into their disease.

### Laying a foundation for a trusting relationship

The encounter was described as important to the creation of an ongoing trusting relationship. The aim was that the relationship should be confidential and that both parties should dare to enter a conversation about mental illness. Encountering an unknown patient was perceived as easier than encountering well-known patients because the nurses had fewer pre-understandings. The ability to read the patient, find confidence in the conversation and establish encounters without time pressure facilitates a trusting relationship.

That you should think about like, trying, as long as they are not aggressive and you don ´t feel threatened, try to get away and talk to them in peace and quiet, cause you will find out much more then, but I guess it all depends on how threatened you feel*.*

Some techniques were mentioned to facilitate trust. These included moving the patient away from a stressful environment, not interrupting, conveying security, and showing interest and respect. If the patient was perceived as having confidence in the nurse, it was easier to talk about anything.

## Discussion

The main findings of this study have been presented in terms of three themes: ‘Lacking trust in the patient and one’s own ability’, ‘Being under internal and external influences’, and ‘Moving towards a genuine nurse-patient relationship’.

Creating a relationship is a crucial part of encountering patients with mental ill-health in prehospital emergency care [23]. The theme ‘Lacking trust in in the patient and one’s own ability’ shows that nurses are aware that they are supposed to manage these complex situations and are striving to increase their awareness so as to improve their confidence and situation awareness in these encounters. They experience difficulty in deciding how firmly they must act in in order to give each patient the best possible care while avoiding triggering the patient and losing control in a potentially dangerous situation. The nurses’ statements that they did not feel safe in these complex situations is in line with another study [24]. In this context, the results of another study conducted on patient aggression showed that their fear is not unfounded for nurses working in ‘PEC’ settings report physical violence and verbal aggression more frequently than psychiatric nurses. Psychiatric nurses also maintain better wellbeing after exposure to patient aggression than prehospital nurses [25]. This finding indicates that more attention should be given to increasing the self-esteem and maintaining the wellbeing of nurses in ‘PEC’. One way of dealing with this issue may be to increase the level of education related to the care of patients with mental illness among nurses in ‘PEC’ [7]. The difficulty of reaching out to patients with mental illness affects the ability to be trustworthy in the relationship. In a stressful situation, the condition needs to be categorized and the nurse has to decide whether the patient is suffering from a physical or mental illness. Holmberg et al. [8] state that lack of time to care for the patient may cause nurses to question their responsibility for caring for the patient. The nurses complained that the information they received before the assignment did not always correspond to what they encountered on arrival. This mismatch created difficulties, but it was found that the primary assessment was easier if the patient could be taken to a calm milieu where the nurse and patient could talk on their own. Rantala et al. [11] argue that nurses’ ability to create a calming atmosphere is important because the situation may go from the patients being insecure to the patients feeling strong enough to take part in decision-making. Taking time to listen to the patient, which includes empathy, silence, attention to both verbal and nonverbal communication, and the ability to be non-judgmental and accepting, has always been considered a crucial component of nursing care [26]. A review study of the experiences of patients with mental health problems shows that they had predominately negative experiences due to poor communication and interpersonal relations [27]. How nurses come across in the acute care setting is essential for those in crisis as their experience impacts on their satisfaction with care [28]. Achieving health can be complex [29]. The results found in resent study is in line with other studies also showing that nurses experience a lack of knowledge when working with patients with mental illness. Similar results due to a need for education are discussed in Yang et al. [30]. They state that education about effective coping strategies and therapeutic nurse-patient communication techniques are needed to minimize the impact of work-related violence on nurses and to improve the quality of health care. This corresponds to nurses in ‘PEC’ who have experience of a dichotomous relationship between somatic care and nurse-patient relationships prioritising the somatic aspects [31]. Hence interventions to support those nurses to understand the holistic aspects of their care is of importance.

The theme ‘Being under internal and external influences’ indicates that nurses’ pre-understandings affect their encounters with the patients. Nurses are influenced by their attitudes. Therefore, it is essential that nurses are aware of their own preconceptions, that is, previous personal and professional experiences, the prevailing culture and traditions, and the way they apply their pre-understanding [32].

Among other things, a nurse’s pre-understanding influences their assessment of the patient. Research indicates that nurses are trying to control their pre-understanding, to interpret and understand several aspects, not only those related to the patient’s physical illness or injury [8]. However, research also indicates the opposite, namely that the focus during assessment is mainly on physical illness or injury. This probably reflects the curriculum, culture and traditions that dominate both education and ‘PEC’ [10]. Consequently, if nurses are more focused on time-critical somatic conditions, without paying attention to patients’ mental illness, they may contribute to increased discomfort and suffering in encounters with patients suffering from mental illness.

The theme ‘Moving towards a genuine nurse–patient relationship’ uncovered an understanding of the encounter with the patient with mental illness as a process towards a deepened and genuine relationship. This relationship is described as being based on the nurses’ ability to be open, instructive, responsive and non-judgemental. This corresponds with earlier research identifying openness as a core value in a nurse–patient relationship [33]. However, earlier research has also shown a condemning attitude towards patients with mental illness in emergency care. Studies also show that suffering mental illness involves a dimension of stigma [34]. These results may serve as a background, adding to the complexity of remaining open towards patients with mental illness in ‘PEC’. This is especially the case when nurses lack competence in mental illness care, and are at risk of seeing such patients as less interesting than patients with somatic conditions [35]. These two factors underline the importance of working with educational interventions and attitudes among personnel working in EMS, to establish a foundation for genuine and caring relationship with patients.

The results also show that in an encounter with patient with mental illness, both parties need courage to enter into conversation. It requires reciprocal trust, earlier described as a core component in a caring relationship [36]. The nurse has to trust the patient and vice versa. Løgstrup argues that receiving trust from another motivates one to assume responsibility [37]. In the present results, this element is found in the sub-theme ‘Laying a foundation for a trusting relationship’. It highlights the importance of the nurse’s performance as a trustworthy professional. Research shows that, from the patient’s perspective, trust is handing over control of the situation to the nurse [12]. As discussed earlier, openness is an important part of enabling this trust. However, the nurses in the present study see themselves as also having a pedagogical role in the relationship. This may introduce an element of conflict into the patient relationship if patients are looking for someone to whom they can hand over responsibility, while nurses see themselves as educators. There is a risk that the patient’s trust may not be understood correctly by the nurses, resulting in a conflict between the nurses’ perceived responsibility and the patients’ needs. Earlier research had indeed shown that patients with mental illness do not think that they are properly cared for in an emergency setting [38]. These findings underscore the need to develop nurses’ ability to understand the nurse–patient relationship from the patient perspective as well.

### Strength and limitations

In order to ensure the rigor and trustworthiness of this study, credibility, dependability, confirmability, and transferability were considered throughout the process [39]. The demographics of the participants in the study show that there was a wide variation in terms of gender, age, educational background and experience. This variety strengthens the study’s findings. However, because all the participating nurses were recruited in the same region of Sweden, there was a risk that their experiences might be coloured by a specific culture. To reduce this risk, the participants were recruited from different independent ambulance stations in the region.

All the authors have a background in clinical emergency care, and the impact of their own pre-understandings must be taken into consideration when reflecting on the *credibility* and d*ependability* of the inductively described patterns in the data. While such impact is unavoidable, attempts were made to minimize it by critical review within the research group (by continuously moving back and forth between the formulated themes and the transcribed data) and at a seminar outside the research group. Moreover, the aim of the chosen method has been to represent data on their own terms without explicit interpretation. Those efforts strengthen the *dependability* and *credibility* of the results. To ensure *confirmability* of the results, quotations from several different respondents’ interviews have been added to the paper to confirm the sub-themes.

*Transferability* is always a challenge in qualitative studies. The efforts to describe the participants, context, data collection and analysis as carefully as possible was done to support the transferability of the results. However, in order to transfer the findings to other settings the results need to be de-contextualized.

A further limitation of this study may be its broad understanding of the concept ‘mental illness’ as including both mild and severe conditions. ‘PEC’ is usually provided to patients without defined diagnoses, and the nature of the care depends on their actual symptoms. This may have contributed to ‘the natural way’ of encountering patients in this setting. The participants shared narratives from a variety of different examples of encounters with patients with mental illness. This, together with their different demographic backgrounds, was judged to contribute to the data’s richness and variation. A more limited understanding of ‘mental illness’ might have risked the inductive approach, leading to less data. However, based on the present results, future studies should be focused on ‘PEC’ and encounters with patients with specific mental illnesses, such as psychosis or suicidal behaviour.

## Conclusion

This study provides insights to nurse’s experiences of encountering patients with mental illness in ‘PEC’. The results show that nurses strive to lay the foundation for a trusting relationship. Simultaneously nurses encountering is characterized by a mistrust and it is influenced by pre-understanding and emotions when they take care for patients. It is essential that patients suffering from mental illness have access to proper ‘PEC’. Therefore, it is important that nurses’ mental health competencies are strengthened and correspond to the requirements in ‘PEC’.

### Implications for practice and future research

The results from this study could be used to develop nurses’ readiness and capability to encounter patients with mental illness and to respond appropriately to the patients somatic and mental care needs. Awareness of nurses encountering will help to advance the teaching and training of nurses in ‘PEC’. To provide appropriate care based on the specific patient, one needs insight in the patient’s lifeworld. This might be a challenge while developing interventions for education in ‘PEC’. Therefore, interventions need to be dynamically designed to caught different aspects of the patient’s lifeworld. Further research that focuses on patients with mental illness in ‘PEC’ should be carried out. It would be valuable to explore what can provide the best possible care and how to maintain and protect the autonomy for patients with mental illness in their encounter with ‘PEC’.

## Data Availability

The datasets used and/or analysed during the current study are available from the corresponding author on reasonable request.

## Abbreviations

PEC: Prehospital emergency care
EMS: Emergency medical service
